# Machine learning models and restricted cubic spline were employed to analyze and predict postoperative ischemic stroke in type A aortic dissection patients

**DOI:** 10.1186/s12872-025-05375-3

**Published:** 2025-12-10

**Authors:** Wenjian Ma, Siji Chen, Yang Zhao, Shuanglei Zhao, Qianxian Li, Yi Hu, Ming Gong

**Affiliations:** 1 Department of Cardiac Surgery, Beijing Anzhen Hospital, Capital Medical University, Beijing, 100029 China; 2 Department of Cadre Ward, The Second Affiliated Hospital of Shenyang Medical College, Shenyang, 110002 Liaoning Province China; 3 Department of Neurology，Beijing Anzhen Hospital,Capital Medical University, 100029 Beijing, China

**Keywords:** Machine learning model, Risk factors, Ischemic stroke, Surgical treatment, Type a aortic dissection

## Abstract

**Background:**

Ischemic stroke remains a devastating postoperative complication in Type A aortic dissection (TAAD) patients, contributing significantly to elevate mortality rates. Identifying reliable predictors for ischemic stroke risk is crucial for implementing timely clinical interventions. This study endeavored to develop and validate a machine learning-based predictive model for ischemic stroke risk stratification in TAAD patients undergoing surgical treatment.

**Methods:**

This retrospective cohort study analyzed 430 TAAD patients who underwent total aortic arch replacement with frozen elephant trunk implantation at Beijing Anzhen Hospital (2015–2021). The cohort was randomly partitioned into training (70%, *n* = 301) and validation (30%, *n* = 129) sets. We selected the top 8 outcome-relevant variables by ranking the intersecting features from the Boruta and LASSO algorithms by their AUC values. seven machine learning models were evaluated through receiver operating characteristic (ROC) curves, decision curve analysis (DCA), Precision-Recall (PR) curve and calibration plots. Model interpretability was enhanced via Shapley Additive Explanations (SHAP), while restricted cubic splines (RCS) elucidated potential non-linear/liner relationships between predictors and result.

**Results:**

The Random Forest model demonstrated superior predictive performance over all other models, with a mean area under the curve (AUC) of 0.810 in the validation cohort and 0.806 in the test cohort. SHAP analysis identified key predictors of postoperative ischemic stroke, including Operative Time, Cardiopulmonary Bypass Time, Intraoperative Blood Loss, Intraoperative Plasma Transfusion ml, age, Myoglobin(Mb), Aortic Cross Clamp Time, and Left Subclavian Artery Perfusion. Additionally, restricted cubic splines (RCS) were independently applied to each continuous variable to examine their nonlinear relationships with the outcome. Finally, we subsequently developed a risk assessment calculator and made it publicly accessible online.

**Conclusion:**

The Random Forest model model demonstrates the best predictive capacity for postoperative ischemic stroke in TAAD patients, offering clinicians a tool for early postoperative risk stratification and personalized therapeutic optimization.

**Supplementary Information:**

The online version contains supplementary material available at 10.1186/s12872-025-05375-3.

Type A aortic dissection (TAAD), a life-threatening cardiovascular emergency disease characterized by end-organ mal-perfusion, exhibits marked time-dependent progression. Studies demonstrated an hourly mortality rate of 1–2% following symptom onset, with emergency surgical mortality decreasing from 5.8% (baseline) to 4.4% post-intervention within 48 h [1]. Despite advancements in modern aortic surgery—including Sun’s procedure [2] that integrated total arch replacement with specialized stent-graft deployment in the descending aorta, central arterial repair techniques, and neuroprotective strategies such as selective cerebral perfusion combined with moderate hypothermic circulatory arrest—the incidence of postoperative neurological complication syndrome (NCs) remains persistently high at 17%−48% [3]. Postoperative NCs following emergency TAAD repair were associated with prolonged intensive care/hospital stays (ischemic stroke: 23 ± 16 days vs. no ischemic stroke: 17 ± 18 days, *P* = 0.021) and morbidity [4, 5]. Ischemic stroke, the most clinically significant NCs, demonstrated a postoperative incidence of 24.8% in our prior study [6]. The development of early, precise predictive models for in-hospital neurological complications carries urgent clinical significance for optimizing decision-making and improving outcomes in this critical population.

While multiple risk factors associated with postoperative ischemic stroke have been identified in existing studies, there remains an urgent need for reliable, data-driven predictive models to systematically identify in-hospital ischemic stroke occurrence following surgical interventions. Furthermore, validated prediction tools specifically targeting ischemic stroke complications in patients undergoing Sun’s procedure (total arch replacement with frozen elephant trunk implantation) are critically lacking in both Chinese and international clinical practice. The development of a precise risk stratification model is therefore imperative to advance preoperative assessment, optimize preventive strategies, and guide therapeutic decision-making, ultimately aiming to reduce postoperative ischemic stroke incidence and improve survival rates through evidence-based interventions.

Machine learning (ML), a specialized subset of artificial intelligence (AI), enables automated extraction of clinically actionable insights for critical tasks including risk stratification, diagnostic classification, and survival prediction. ML algorithms have thus emerged as indispensable tools in biomedical research [7], demonstrating capabilities to identify latent patterns within complex datasets and generate predictive outputs through advanced feature engineering [8]. Comparative analyses reveal ML’s superior performance metrics over conventional statistical methods, with successful clinical implementations [9] and real-time treatment optimization [10].

## Materials and methods

### Data source

This retrospective study enrolled patients diagnosed with TAAD who underwent total aortic arch replacement with frozen elephant trunk implantation (Sun’s procedure) at the Cardiac Surgery Center of Beijing Anzhen Hospital, Capital Medical University, between 2015 and 2021. A total of 430 consecutive cases with complete perioperative data were included. The primary endpoint was postoperative ischemic stroke occurrence. Diagnosis adhered to the 2014 ESC guidelines [11], incorporating Stanford classification and confirmatory imaging magnetic resonance angiography (MRA) or computed tomography angiography (CTA). Exclusion criteria comprised:1.Non-surgically managed TAAD 2.Concurrent malignancies with limited life expectancy 3.Acute myocardial infarction secondary to severe myocardial mal-perfusion 4.Incomplete medical records. The diagnosis of postoperative ischemic stroke was rigorously defined as any new, focal neurological deficit of cerebrovascular origin, with symptoms persisting for ≥ 24 h and confirmed by neuroimaging, in accordance with internationally recognized guidelines such as the American Heart Association/American Stroke Association (AHA/ASA) 2019 Guidelines [12]. Diagnoses were established within 30 days following the index surgical procedure. All cases required neuroimaging confirmation via magnetic resonance imaging (MRI) with diffusion-weighted imaging (DWI) or computed tomography perfusion (CTP) to delineate the infarct core. Both major (e.g., causing significant functional impairment, typically NIHSS score ≥ 5) and minor strokes (e.g., NIHSS score ≤ 3 or 5, depending on study criteria) were included, provided the deficit persisted for ≥ 24 h [13]. The cohort was stratified into a training set (70%, *n* = 301) and a validation set (30%, *n* = 129) using stratified random sampling to balance baseline characteristics and mitigate overfitting risks. The study protocol was approved by the Institutional Review Board of Beijing Anzhen Hospital (Approval No: 2025124X), and the research strictly adhered to the ethical principles of the Declaration of Helsinki.

### Features extraction

The extracted variables encompassed demographic characteristics (age, BMI), clinical profiles (hypertension, history of coronary artery disease, diabetes, smoking/alcohol use, history of cerebrovascular disease, renal insufficiency, prior cardiac surgery, NYHA functional class, acute renal dysfunction), surgical parameters (intraoperative blood loss, cardiopulmonary bypass duration, deep hypothermic circulatory arrest time, aortic cross-clamp time exceeding 3 h), and ultrasonographic findings including dissection involvement of the left subclavian artery, innominate artery, left common carotid artery, along with true/false lumen perfusion patterns. Laboratory biomarkers comprised hepatic function indices (ALT, AST), coagulation markers (D-dimer), myocardial injury indicators (myoglobin), and renal function tests (serum creatinine). Blood samples were collected within 24 h of admission, with the initial measurement utilized for variables requiring repeated assessments to ensure temporal consistency and minimize intervention-related confounding.

### Surgical technique

Sun’s procedure refers to total arch replacement using a four-branched vascular graft combined with specialized stent-graft implantation in the descending aorta, as technically detailed in the Annals of Thoracic Surgery [14]. Briefly, the procedure was performed under moderate hypothermia at 25 °C with circulatory arrest. Cardiopulmonary bypass was established via right axillary artery cannulation, incorporating selective antegrade cerebral perfusion. The surgical steps included: stent-graft deployment in the descending aorta and total arch replacement with a four-branched graft. A specific sequence of vascular reconstruction was followed: proximal descending aorta anastomosis first, followed by the left carotid artery, ascending aorta, left subclavian artery, and finally the celiac artery. Early rewarming and reperfusion were initiated after completion of the distal anastomosis to minimize cerebral and coronary ischemic time.

### Model construction and validation

**Fig. 1:**
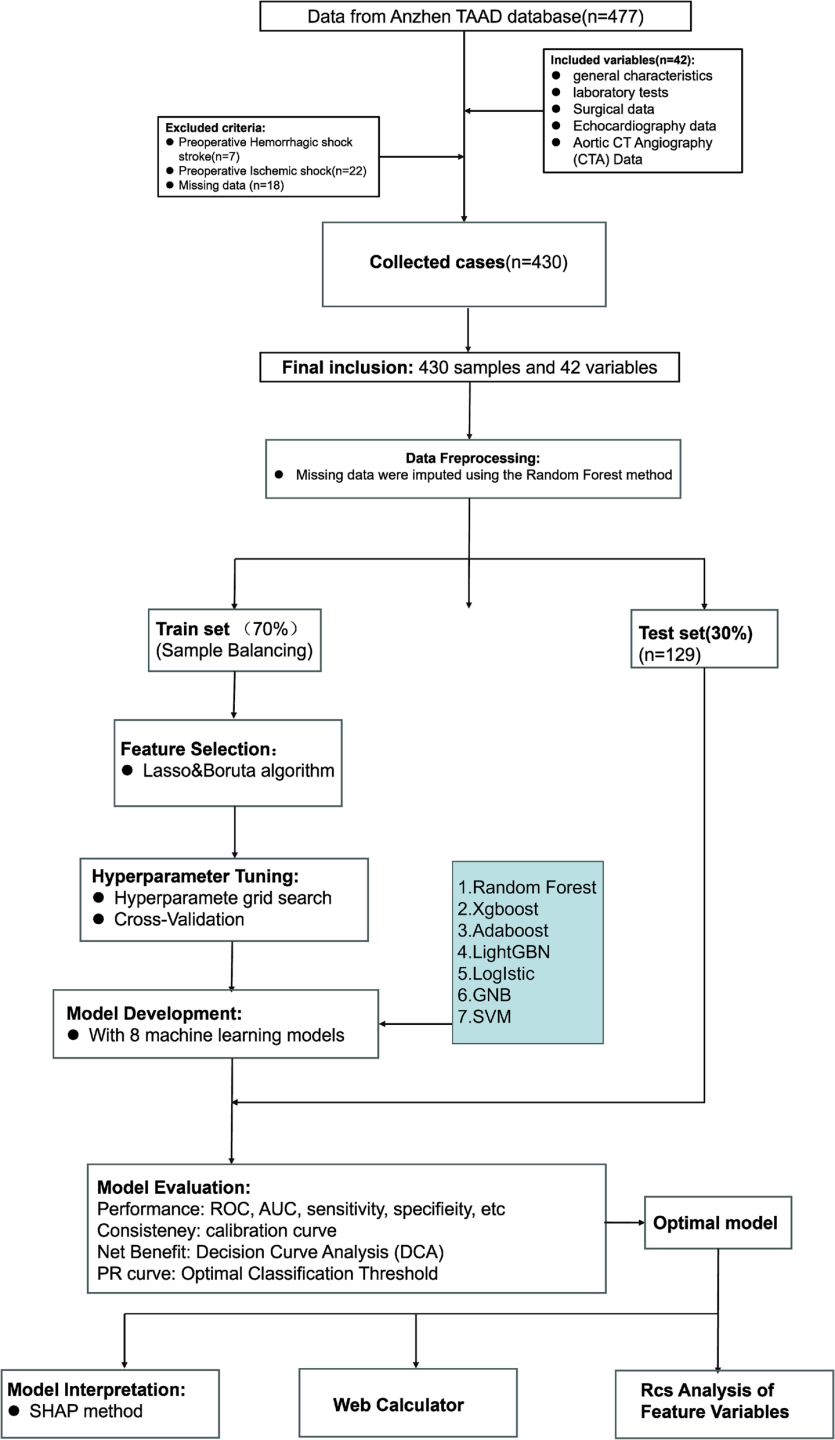
Flowchart of model construction

**Fig. 2:**
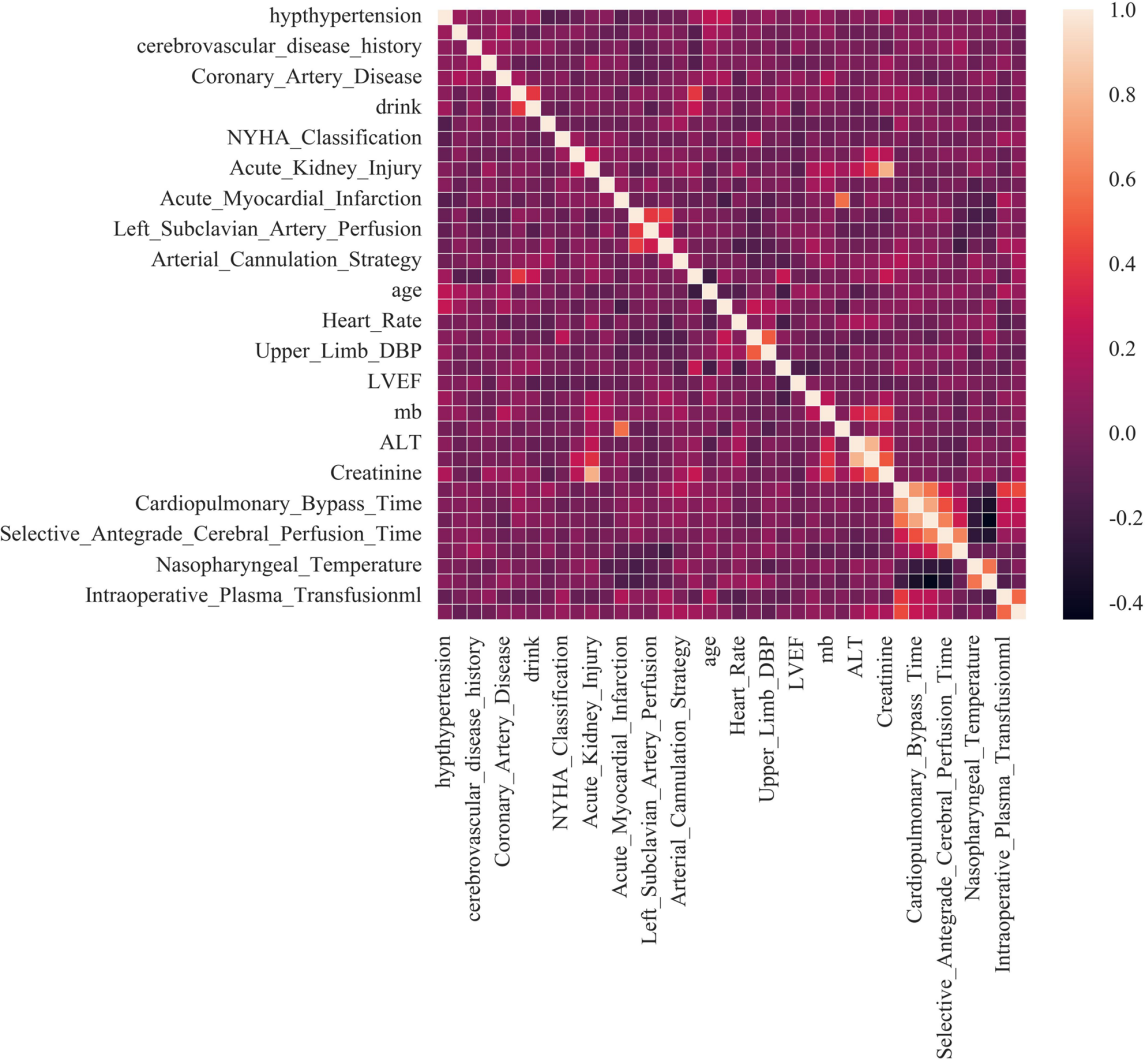
The correlation heatmap illustrated the correlation between each factor

Figure 1 displayed the concise flowchart of predictive model construction and validation. Spearman correlation analysis was employed to investigate the interrelationships among the variables. The correlation heatmap (Fig. 2) illustrated the correlation between each factor, like the correlation coefficient between N and T is 0.36, which was less than 0.5, indicating a weak correlation, and the others are also weak correlations. Collinearity arises when two or more predictor variables exhibit strong correlation, thereby complicating the assessment of each variable’s distinct contribution to the outcome. So, we selected the most readily available variables among the collinear variables for further analysis.

**Fig. 3:**
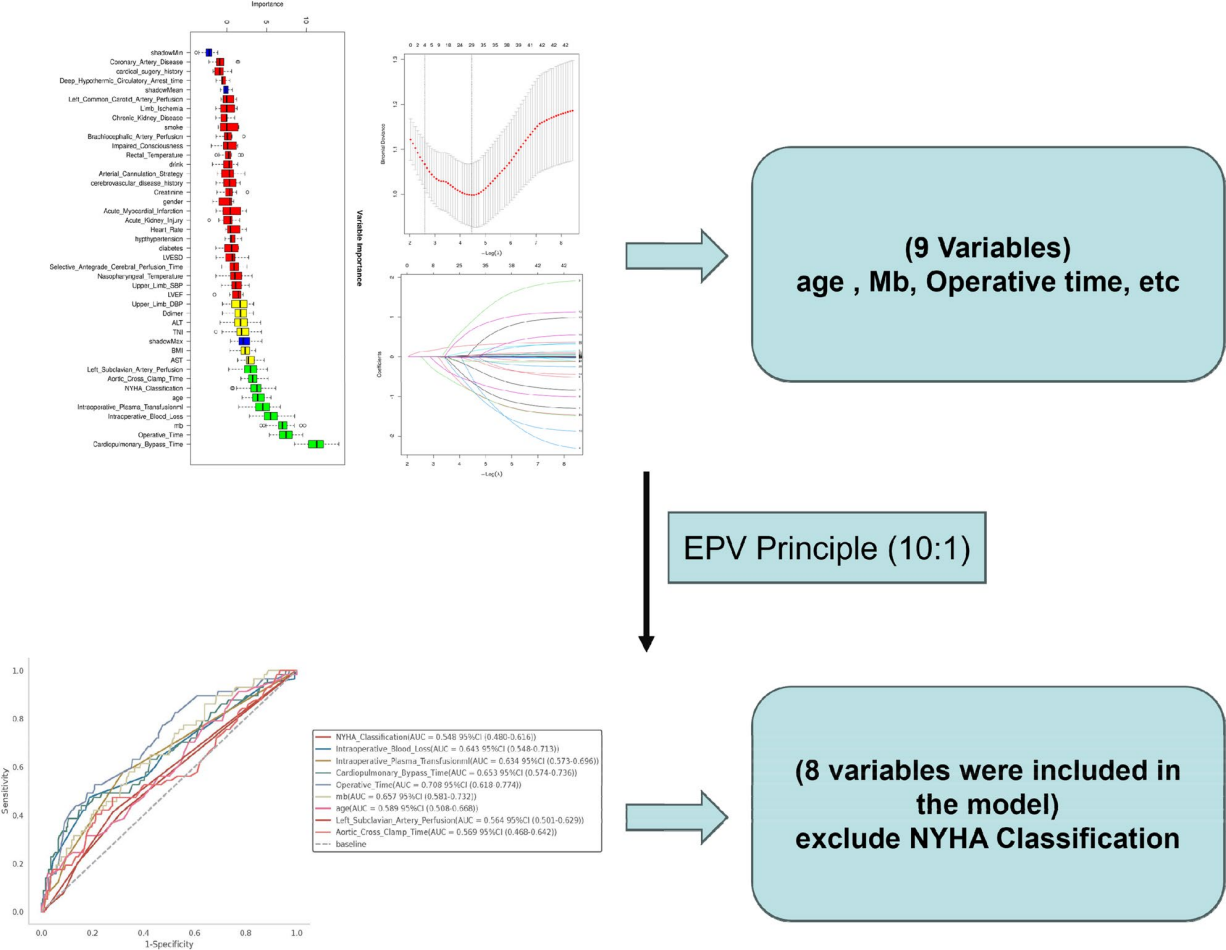
In the training set, we employed a two-step strategy for variable selection to mitigate multicollinearity and identify the most outcome-relevant features. First, LASSO regression was applied to reduce variable collinearity. Second, the Boruta algorithm was used to select variables most associated with the outcome. The intersection of results from these two methods yielded 9 candidate variables. To prevent overfitting given the 80 positive events in the training set (adhering to the Events Per Variable (EPV) principle), we excluded the variable with the lowest AUC(NYHA Classification). Consequently, 8 variables were ultimately incorporated into the final mod

In the screening of potential risk factors, we employed a strategy of parallel analysis using both LASSO regression and the Boruta algorithm, followed by taking the intersection of their results. LASSO achieves efficient feature selection by applying L1 regularization to compress the coefficients of weakly relevant features to zero, excelling in addressing multicollinearity and constructing parsimonious linear models. The Boruta algorithm, through the creation of “shadow features” and comparative random forest importance assessments, enables unbiased identification of all relevant features, including those with nonlinear relationships. After independent execution of both methods, we took the intersection of their selected feature sets as the final cohort. This intersection underwent dual validation through both model regularization-based stringent screening and comprehensive statistical importance-based detection, ensuring both feature selection simplicity and preservation of significant signals. The ultimately identified risk factors thus demonstrate enhanced credibility and interpretability. Subsequently, we obtained 9 variables and ranked them by their area under the curve (AUC) values. To comply with the Events Per Variable (EPV) principle and reduce model overfitting, the variable with the lowest AUC was excluded, resulting in 8 variables being incorporated into the final model construction (Fig. 3).

The selected significant variables were incorporated into ten distinct machine learning algorithms for model construction, including Gaussian Naive Bayes(GNB), Logistic Regression (LR), Support Vector Machine (SVM), Random Forest (RF), Extreme Gradient Boosting (XGBoost), Adaptive Boosting (AdaBoost), Light Gradient Boosting Machine (LightGBM). On the training set, we employed grid search for automatic hyperparameter optimization, using 10-fold cross-validation for internal model validation. Model performance was evaluated by selecting the algorithm achieving the highest AUC, with supplementary assessments of discriminatory power conducted through sensitivity, specificity, accuracy, and F1-score metrics. DCA curve quantified clinical utility by estimating net benefit across probability thresholds. The PR curve evaluates a model’s ability to identify positive samples across different thresholds, particularly valuable for class-imbalanced datasets.

The interpretability of machine learning models remains a significant challenge in clinical applications. To elucidate how individual features contribute to predictions in our best-performing black-box model, we employed SHAP values—a game theory-based approach that quantifies each feature’s impact on model outputs by treating features as collaborative players. SHAP fairly attributes predictive contributions to each variable, enabling both global interpretation through mean absolute SHAP value ranking and local explanation via individual prediction decomposition. Feature importance was determined by calculating the mean absolute SHAP value across all observations. Additionally, we visualized force plots and summary plots to delineate the directional effects of key predictors.

### Statistical analysis

The missing values for all variables in this study were less than 10%. To preserve data integrity, we performed data imputation using the Random Forest method. Statistical description: All Statistical analyses were performed using R version 4.2.3 and python version 3.11.4.Acknowledgments༚This work is supported by Extreme Smart Analysis platform (https://www.xsmartanalysis.com/). Normally distributed continuous variables were presented as mean ± standard deviation (SD), with between-group comparisons performed using Student’s t-test. Non-normally distributed variables were expressed as median (interquartile range, IQR) and analyzed via the Mann-Whitney U test. Categorical variables were reported as counts (percentages), with group differences assessed using Pearson’s chi-square test or Fisher’s exact test as appropriate for cell frequencies.

## Result

### Baseline characteristics

Following rigorous screening, this study enrolled 430 patients, with 301 allocated to the training set and 129 to the testing set. The postoperative ischemic stroke incidence was 17% in both cohorts. The overall cohort demonstrated a median age of 48.0 years (38.0–55.0), with a male predominance (22.2% female vs. 77.7% male). Table 1 details baseline characteristics including demographic parameters, vital signs, and laboratory indices. Comparative analysis revealed statistically significant differences (*P* < 0.05) between ischemic stroke and non-ischemic stroke groups: Preoperative Factors: Ischemic stroke patients were older (median age 50 vs. 47 years, *P* = 0.035) with greater prevalence of cerebrovascular disease history (7.0% vs. 1.6%, *P* = 0.045).Biochemical Profile: Elevated myoglobin levels (median 33.5vs. 49.2 ng/mL, *P* < 0.001) and impaired hepatic function (AST: 22 vs. 27U/L, *P* = 0.004) were observed in ischemic stroke cases. Intraoperative Metrics: Prolonged cardiopulmonary bypass time (CPBT: 201 vs. 215 min, *P* < 0.001) and increased intraoperative blood loss (IBV: 1200 vs. 1500mL, *P* < 0.001). Femoro-axillary arterial cannulation is associated with a higher risk of postoperative cerebral infarction(*P* = 0.025).

**Table 1:** Patient Demographics and Baseline Characteristics in the Training Cohort

Characteristic	[ALL] *N*=301	No ischemic stroke *N*=244	ischemic stroke *N*=57	*p*
Age (year), Median [Q1-Q3]	48.000 [38.000;55.000]	47.500 [37.000;55.000]	50.000 [43.000;58.000]	0.035
BMI (kg/m²), Median [Q1-Q3]	26.235 [24.221;29.388]	26.185 [24.201;29.297]	27.682 [24.221;29.412]	0.250
Heart Rate (bpm), Median [Q1-Q3]	84.000 [74.000;94.000]	85.000 [74.000;93.000]	82.000 [75.000;99.000]	0.560
Upper Limb SBP (mmHg), Median [Q1-Q3]	128.000 [117.000;145.000]	129.000 [117.000;146.000]	127.000 [122.000;140.000]	0.754
Upper Limb DBP (mmHg), Mean (SD)	68.439 (14.236)	67.795 (14.199)	71.193 (14.187)	0.107
LVESD (mm), Mean (SD)	49.530 (6.449)	49.740 (6.324)	48.631 (6.947)	0.273
LVEF (%), Median [Q1-Q3]	62.000 [59.000;66.000]	62.000 [60.000;66.000]	60.000 [58.000;65.000]	0.315
hypertension, *N* (%):				1.000
No	52 (17.276%)	42 (17.213%)	10 (17.544%)	
Yes	249 (82.724%)	202 (82.787%)	47 (82.456%)	
diabetes, *N* (%):				0.386
No	281 (93.355%)	226 (92.623%)	55 (96.491%)	
Yes	20 (6.645%)	18 (7.377%)	2 (3.509%)	
Cerebrovascular disease history, *N* (%):				0.045
No	293 (97.342%)	240 (98.361%)	53 (92.982%)	
Yes	8 (2.658%)	4 (1.639%)	4 (7.018%)	
Chronic Kidney Disease, *N* (%):				1.000
No	296 (98.339%)	240 (98.361%)	56 (98.246%)	
Yes	5 (1.661%)	4 (1.639%)	1 (1.754%)	
Coronary Artery Disease, *N* (%):				0.405
No	278 (92.359%)	227 (93.033%)	51 (89.474%)	
Yes	23 (7.641%)	17 (6.967%)	6 (10.526%)	
smoke, *N* (%):				0.657
No	169 (56.146%)	135 (55.328%)	34 (59.649%)	
Yes	132 (43.854%)	109 (44.672%)	23 (40.351%)	
drink, *N* (%):				1.000
No	225 (74.751%)	182 (74.590%)	43 (75.439%)	
Yes	76 (25.249%)	62 (25.410%)	14 (24.561%)	
Cardical sugery history, *N* (%):				0.705
No	289 (96.013%)	235 (96.311%)	54 (94.737%)	
Yes	12 (3.987%)	9 (3.689%)	3 (5.263%)	
NYHA Classification, *N* (%):				0.374
2	62 (20.598%)	52 (21.311%)	10 (17.544%)	
3	33 (10.963%)	30 (12.295%)	3 (5.263%)	
4	24 (7.973%)	19 (7.787%)	5 (8.772%)	
Impaired Consciousness, *N* (%):				0.318
No	286 (95.017%)	230 (94.262%)	56 (98.246%)	
Yes	15 (4.983%)	14 (5.738%)	1 (1.754%)	
Acute Kidney Injury, *N* (%):				0.166
No	271 (90.033%)	223 (91.393%)	48 (84.211%)	
Yes	30 (9.967%)	21 (8.607%)	9 (15.789%)	
Limb Ischemia, *N* (%):				0.338
No	267 (88.704%)	219 (89.754%)	48 (84.211%)	
Yes	34 (11.296%)	25 (10.246%)	9 (15.789%)	
Acute Myocardial Infarction, *N* (%):				0.152
No	279 (92.691%)	229 (93.852%)	50 (87.719%)	
Yes	22 (7.309%)	15 (6.148%)	7 (12.281%)	
Left Common Carotid Artery Perfusion, *N* (%):				0.705
No	207 (68.771%)	166 (68.033%)	41 (71.930%)	
True	93 (30.897%)	77 (31.557%)	16 (28.070%)	
False	1 (0.332%)	1 (0.410%)	0 (0.000%)	
Left Subclavian Artery Perfusion, *N* (%):				0.074
No	184 (61.130%)	143 (58.607%)	41 (71.930%)	
True	114 (37.874%)	99 (40.574%)	15 (26.316%)	
False	3 (0.997%)	2 (0.820%)	1 (1.754%)	
Brachiocephalic Artery Perfusion, *N* (%):				0.387
No	159 (52.824%)	133 (54.508%)	26 (45.614%)	
True	138 (45.847%)	108 (44.262%)	30 (52.632%)	
False	4 (1.329%)	3 (1.230%)	1 (1.754%)	
Arterial Cannulation Strategy, *N* (%):				0.025
Axillary Artery	193 (64.120%)	165 (67.623%)	28 (49.123%)	
Femoral Artery	31 (10.299%)	24 (9.836%)	7 (12.281%)	
Axillary-Femoral Artery	77 (25.581%)	55 (22.541%)	22 (38.596%)	
gender, *N* (%):				0.947
female	67 (22.259%)	55 (22.541%)	12 (21.053%)	
male	234 (77.741%)	189 (77.459%)	45 (78.947%)	
D-dimer (μg/mL), Median [Q1-Q3]	2320.000 [1032.000;4325.000]	2301.500 [1042.500;3466.500]	2351.000 [1029.000;8468.000]	0.338
Mb (ng/mL, Median [Q1-Q3]	34.700 [21.500;68.000]	33.500 [20.725;60.696]	49.200 [30.500;145.900]	<0.001
TNI (ng/mL, Median [Q1-Q3]	0.010 [0.010;0.050]	0.010 [0.010;0.050]	0.030 [0.010;0.110]	0.079
ALT (U/L), Median [Q1-Q3]	22.000 [16.000;36.000]	22.000 [15.750;34.000]	24.000 [17.000;40.000]	0.151
AST (U/L), Median [Q1-Q3]	22.000 [18.000;32.000]	22.000 [17.000;30.000]	27.000 [20.000;36.000]	0.004
Creatinine (μmol/L), Median [Q1-Q3]	81.000 [68.600;100.100]	80.350 [68.225;98.425]	83.800 [72.200;106.700]	0.126
Operative Time (hour), Median [Q1-Q3]	7.500 [6.500;8.300]	7.200 [6.371;8.000]	8.200 [7.300;9.400]	<0.001
Cardiopulmonary Bypass Time (min), Median [Q1-Q3]	203.000 [182.000;232.000]	201.000 [180.000;223.250]	215.000 [192.000;268.000]	<0.001
Aortic Cross Clamp Time (min), Median [Q1-Q3]	112.000 [96.000;137.000]	111.000 [95.750;132.000]	119.000 [98.000;149.000]	0.105
Selective Antegrade Cerebral Perfusion Time (min), Median [Q1-Q3]	35.260 [29.000;44.000]	35.000 [30.000;44.000]	37.000 [28.000;47.000]	0.469
Deep Hypothermic Circulatory Arrest time (min), Median [Q1-Q3]	22.000 [18.000;29.000]	22.000 [18.000;28.000]	25.000 [17.000;30.000]	0.405
Nasopharyngeal Temperature (°C), Median [Q1-Q3]	24.200 [23.200;24.800]	24.200 [23.200;24.700]	24.100 [22.900;24.800]	0.834
Rectal Temperature (°C), Median [Q1-Q3]	25.600 [24.700;26.500]	25.600 [24.700;26.500]	25.400 [24.700;26.300]	0.374
Intraoperative Plasma Transfusion (ml), Median [Q1-Q3]	0.000 [0.000;500.000]	0.000 [0.000;400.000]	400.000 [0.000;600.000]	<0.001
Intraoperative Blood Loss (mL), Median [Q1-Q3]	1300.000 [1000.000;1500.000]	1200.000 [1000.000;1500.000]	1500.000 [1200.000;2000.000]	0.001

*Abbreviations*: *NYHA* Classification, New York Heart Association (NYHA) Classification, *SBP* systolic blood pressure, *DBP* diastolic blood pressure, *LVEF* Left Ventricular Ejection Fraction, *Mb* Myoglobin, *TNI *Troponin I, *ALT* Alanine Aminotransferase, *AST* Aspartate Aminotransferase, *LVEDD* Left Ventricular End-Diastolic Diameter. (In the table, "No" indicates that the artery was not involved by the dissection. "True" signifies perfusion from the true lumen, and "False" signifies perfusion from the false lumen)

### Performance comparison of seven ML algorithms

**Fig. 4:**
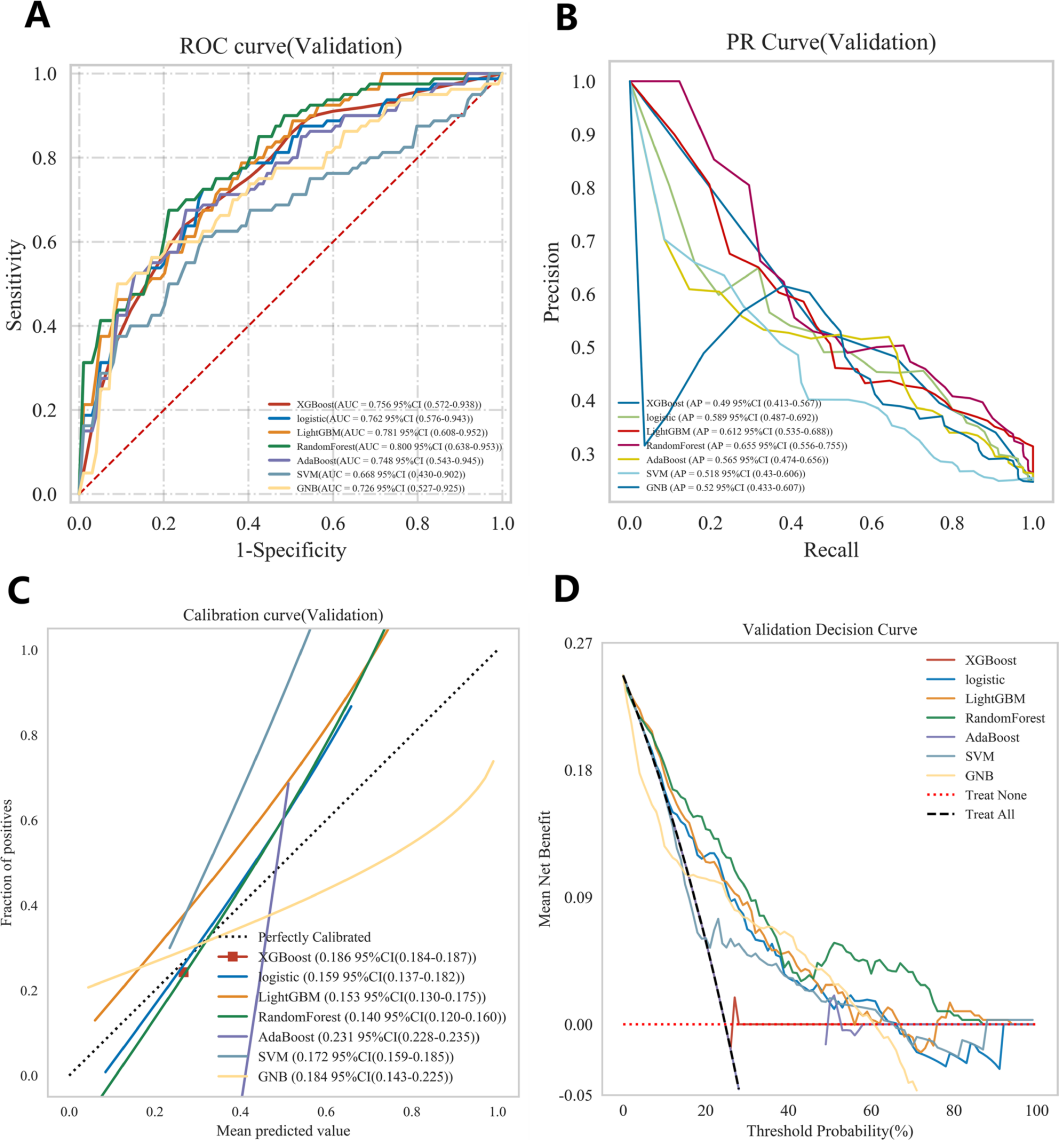
(**A**) Receiver Operating Characteristic (ROC) curves for the validation cohorts. The curves illustrate the discriminatory ability of different predictive models, with the area under the ROC curve (AUC) values displayed for seven models. (**B**) The Precision-Recall (PR) curve demonstrates the model’s performance in classifying the positive class, particularly under class imbalance. (**C**) Calibration Curves for the validation cohorts. The curves verify the degree of conformity between the apparent line and the ideal line. (**D**) Decision Curve Analysis (DCA) for the validation cohorts. The curves show the net benefit of each model across various threshold probabilities, comparing them with the “Treat All” and “Treat None” strategies, indicating their potential clinical utility

**Fig. 5:**
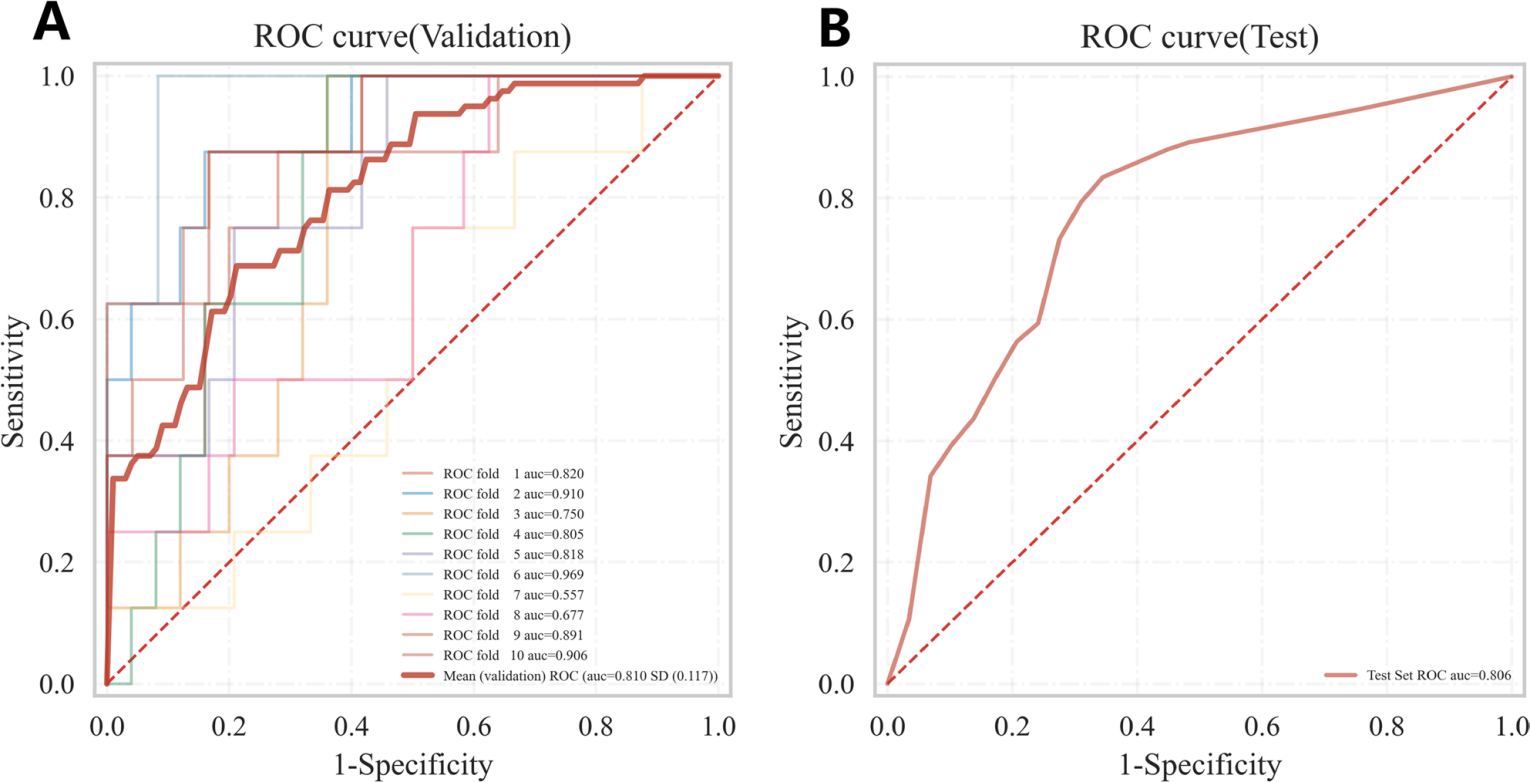
ROC Curves for Internal and External Validation. (**A**) Average ROC from 10-fold cross-validation (internal). (**B**) ROC on the independent test set (external)

**Fig. 6:**
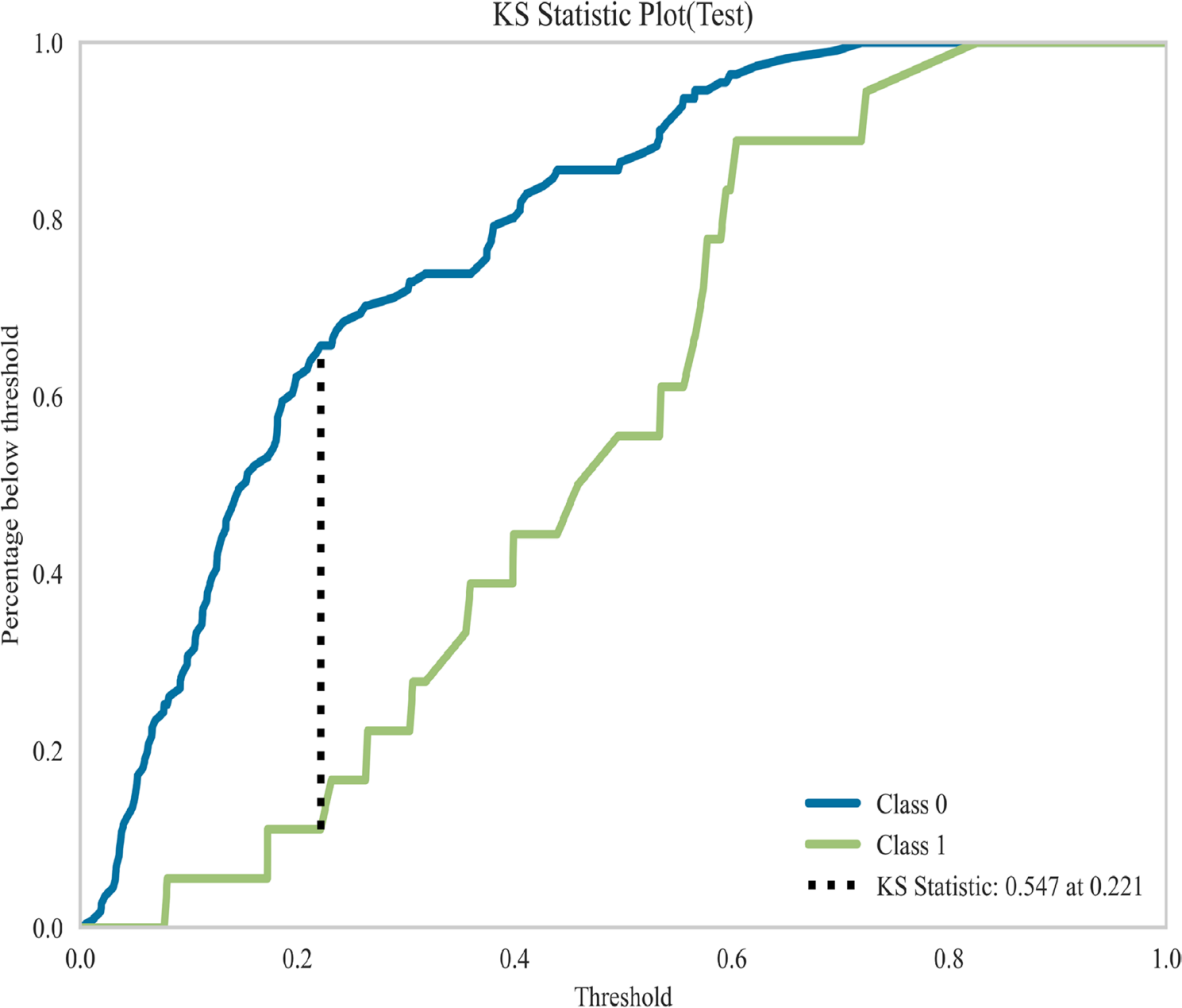
The Kolmogorov-Smirnov (KS) curve evaluates a model’s discriminatory power by measuring the maximum separation between the cumulative distribution functions of two classes. As shown in the figure, the KS statistic of 0.547 indicates that the model demonstrates excellent performance in distinguishing between positive and negative samples

**Fig. 7:**
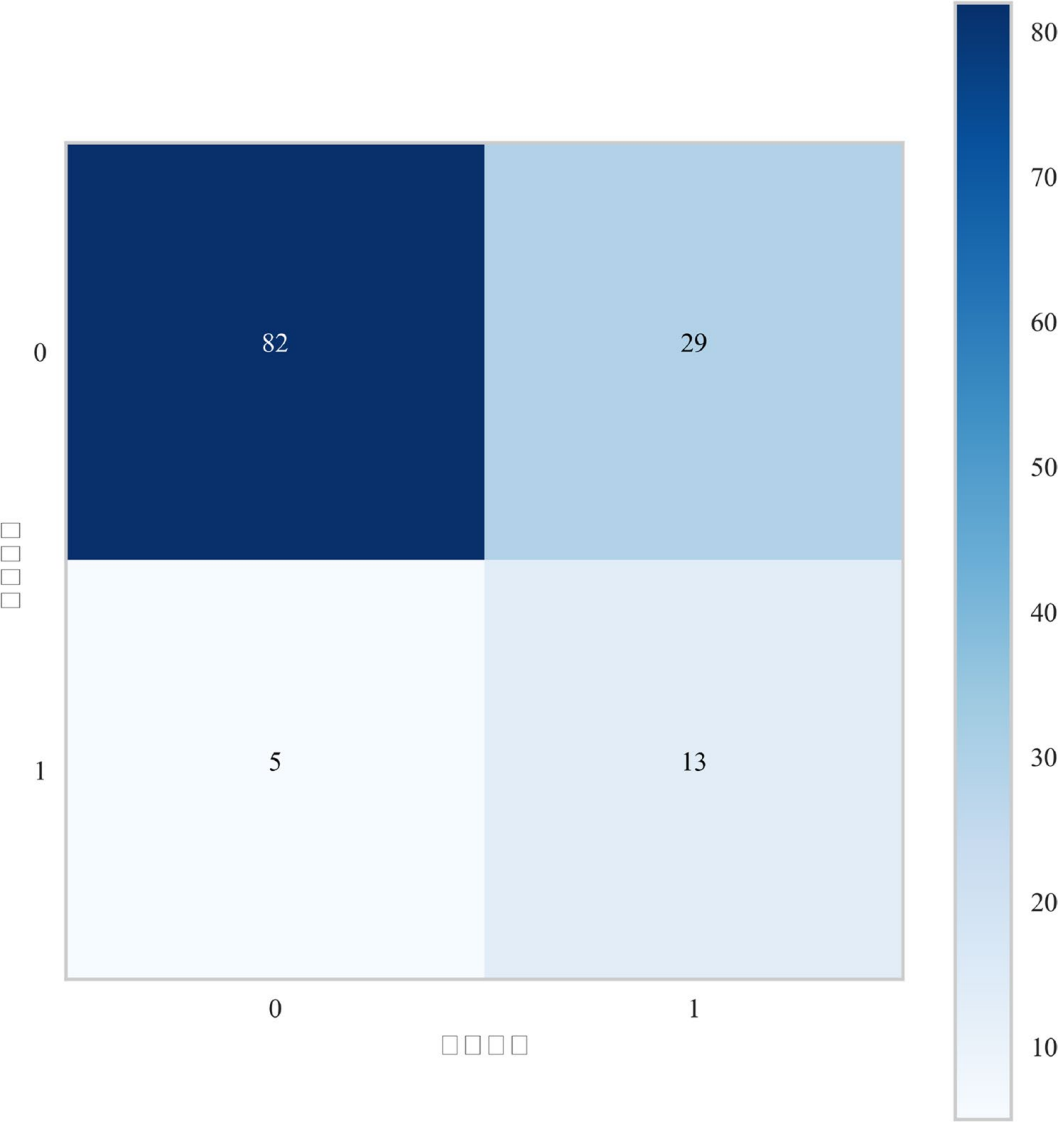
Confusion Matrix on the Test Set

This study compared the performance of seven machine learning models in predicting postoperative ischemic stroke in patients with type A aortic dissection, presenting their results on receiver operating characteristic (ROC) curve, Decision Curve Analysis (DCA), cross-validation (CV) curve, and precision-recall(PR) curves (Fig. 4A-D). In internal validation, the Random Forest model demonstrated outstanding predictive performance with a mean area under the curve obtained through 10-fold cross-validation was 0.810, and it maintained excellent performance in the test cohort with an area under the curve of 0.806, indicating stable and accurate predictive capability (Fig. 5A). Other models achieved the following area under the curve values: XGBoost 0.756, logistic regression 0.762, AdaBoost 0.748, and LightGBM 0.781, while the support vector machine model performed relatively poorest with an area under the curve of only 0.668 (Fig. 5A). Decision curve analysis further confirmed that the Random Forest model provided the highest net benefit across most threshold probability ranges, particularly in intermediate probabilities, highlighting its superiority for clinical application (Fig. 4B). Detailed performance metrics for all models, including sensitivity, specificity, area under the curve, accuracy, and F1-score, are provided in Table 2. The Random Forest model achieved the best comprehensive discriminative ability with an accuracy of 0.772, specificity of 0.840, and F1-score of 0.554. Figure 6 displays the Kolmogorov-Smirnov curve for this model on the test set, where a KS statistic of 0.547 indicates excellent performance in distinguishing positive and negative samples. Figure 7 further presents the corresponding confusion matrix for the Random Forest model.

**Table 2:** Comparison of validation Cohort Results of the Machine Learning Models

Model	AUC (95%CI)	cutoff(95%CI)	Accuracy (95%CI)	Sensitivity (95%CI)	Specificity (95%CI)	Positive Predictive Value (95% CI)	Negative Predictive Value (95%CI)	F1(95%CI)	Kappa(95%CI)
XGBoost	0.756 (0.572–0.938.572.938)	0.267(0.267-0.267.267.267)	0.683(0.624–0.741.624.741)	0.725(0.637–0.813.637.813)	0.669(0.587–0.751.587.751)	0.436(0.372–0.501.372.501)	0.883(0.853–0.913.853.913)	0.534(0.476–0.593.476.593)	0.325(0.233–0.416.233.416)
logistic	0.762 (0.576–0.943.576.943)	0.228(0.220–0.237.220.237)	0.7(0.644–0.757.644.757)	0.738(0.625–0.850.625.850)	0.688(0.632–0.745.632.745)	0.443(0.375–0.510.375.510)	0.89(0.845–0.935.845.935)	0.549(0.471–0.627.471.627)	0.348(0.233–0.462.233.462)
LightGBM	0.781 (0.608–0.952.608.952)	0.293(0.264–0.322.264.322)	0.747(0.693–0.801.693.801)	0.637(0.509–0.766.509.766)	0.783(0.712–0.854.712.854)	0.513(0.430–0.596.430.596)	0.872(0.831–0.914.831.914)	0.551(0.469–0.633.469.633)	0.384(0.271–0.496.271.496)
Random Forest	0.800 (0.638–0.953.638.953)	0.357(0.341–0.373.341.373)	0.772(0.722–0.821.722.821)	0.562(0.440–0.685.440.685)	0.84(0.802–0.879.802.879)	0.537(0.434–0.640.434.640)	0.856(0.819–0.894.819.894)	0.544(0.438–0.649.438.649)	0.393(0.257–0.529.257.529)
AdaBoost	0.748 (0.543–0.945.543.945)	0.49(0.488–0.491.488.491)	0.713(0.649–0.778.649.778)	0.625(0.510–0.740.510.740)	0.742(0.677–0.808.677.808)	0.453(0.356–0.550.356.550)	0.858(0.818–0.897.818.897)	0.521(0.419–0.623.419.623)	0.328(0.186–0.470.186.470)
SVM	0.668 (0.430–0.902.430.902)	0.229(0.224–0.233.224.233)	0.704(0.653–0.755.653.755)	0.5(0.363–0.637.363.637)	0.771(0.697–0.845.697.845)	0.432(0.360–0.505.360.505)	0.829(0.792–0.866.792.866)	0.44(0.347–0.533.347.533)	0.25(0.141–0.360.141.360)
GNB	0.726 (0.527–0.925.527.925)	0.122(0.068–0.175.068.175)	0.686(0.625–0.746.625.746)	0.675(0.503–0.847.503.847)	0.69(0.606–0.773.606.773)	0.408(0.326–0.489.326.489)	0.876(0.827–0.926.827.926)	0.498(0.387–0.609.387.609)	0.293(0.157–0.428.157.428)

### Interpretability analysis

**Fig. 8:**
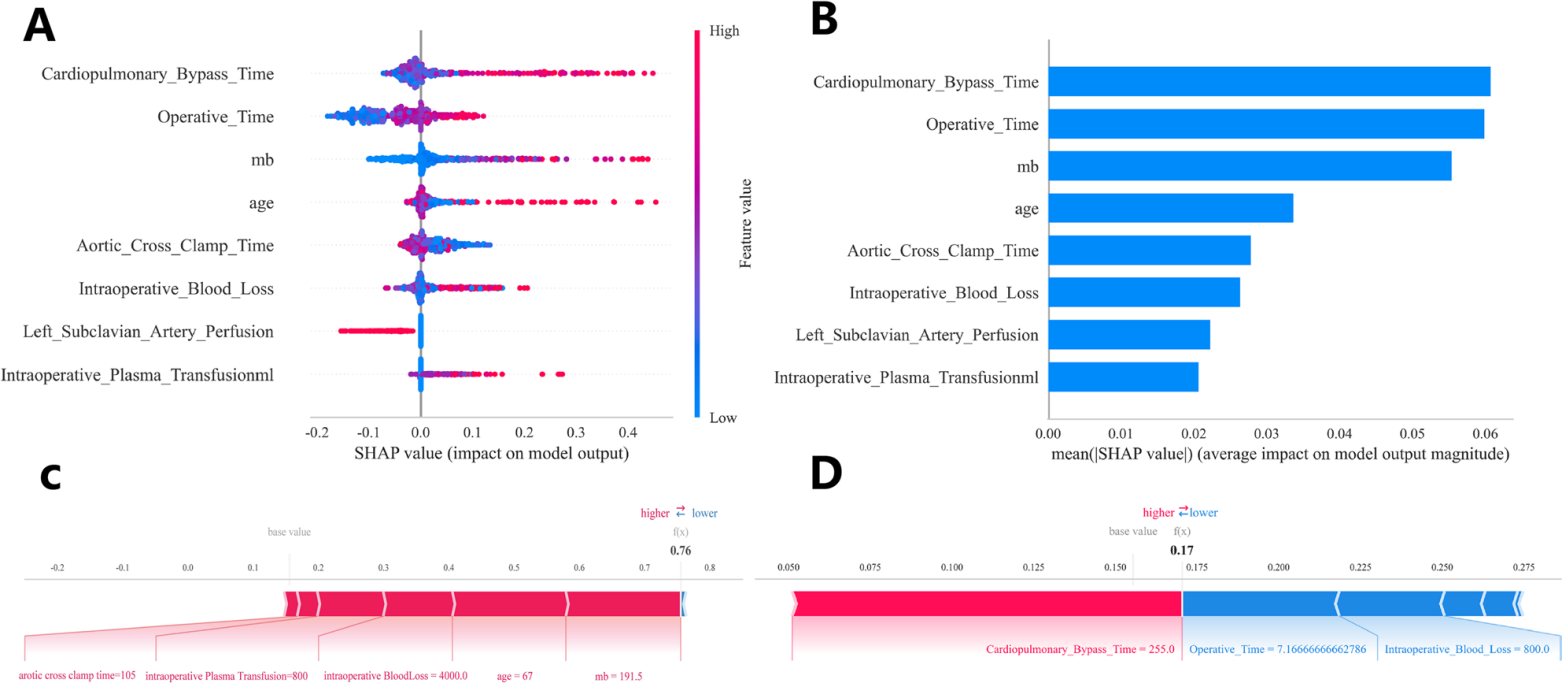
SHAP (Shapley Additive explanations) analysis of the model. (**A**) SHAP summary plot visualizing the distribution of SHAP values for each feature. Each dot represents an individual data point, with the x-axis showing the SHAP value (feature’s impact on the model output) and the color representing the feature value (red for higher values blue for lower values). (**B**) Bar plot showing the mean SHAP values for the top features ranked by their contribution to stroke prediction in TAAD patients. (**C** & **D**):two examples of SHAP predictions for the purpose of demonstration

To gain deeper insights into the relationship between the model and the data, we employed SHAP analysis to provide an intuitive interpretation of the top-performing Random Forest (RF) model, elucidating how these variables influence the occurrence of ischemic stroke in the model. Figure 8A presents the evaluation results of eight important risk factors through SHAP values. The SHAP value on the x-axis serves as a unified metric to determine the impact of specific features on the model’s predictions. In each feature importance row, individual participants’ contributions to the outcomes are represented as color-coded dots, with blue and red points indicating high-risk and low-risk values of the feature, respectively. Figure 8B displays the ranking of important features in the model, where their positions on the y-axis reflect their predictive importance. The analysis revealed significant correlations between cardiopulmonary bypass time, intraoperative blood loss, age, Mb, operation time, aortic clamp time, intraoperative plasma transfusion volume, as well as left subclavian artery involvement by the dissection, and the predicted probability of ischemic stroke. Furthermore, we provided prediction examples for both ischemic stroke-free (Fig. 8C) and ischemic stroke cases (Fig. 8D) to demonstrate the interpretability of the model.

### Linear relationship between continuous variables in the model and postoperative ischemic stroke in TAAD patients

**Fig. 9:**
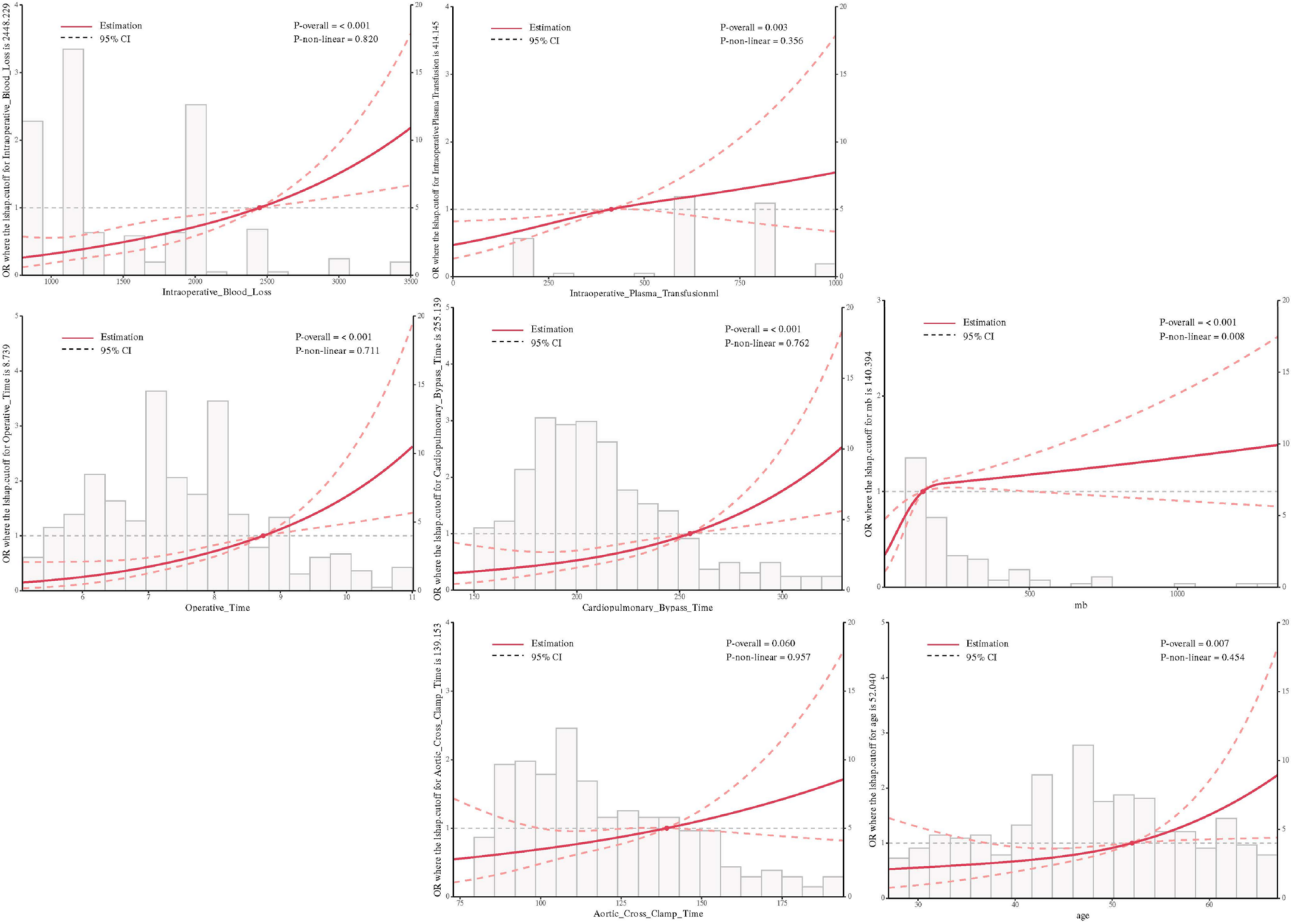
Restricted cubic spline (RCS) analysis of the continuous variables included in the model

We employed restricted cubic spline (RCS) curves to investigate the nonlinear relationships between continuous variables and postoperative ischemic stroke in patients with type A aortic dissection (Fig. 9). The results demonstrated that cardiopulmonary bypass time, intraoperative blood loss, age, myoglobin, operative time, aortic cross-clamp time, and intraoperative plasma transfusion volume all showed statistically significant associations with the outcome. Using the RCS curves, we further identified thresholds where the odds ratio exceeded 1: ischemic stroke risk significantly increased when cardiopulmonary bypass time exceeded 255.1 min, intraoperative blood loss surpassed 2442.2 ml, operation time exceeded 8.3 h, intraoperative plasma transfusion volume exceeded 434.1 ml, myoglobin levels exceeded 140.3 ng/mL, and age was above 52 years.

### Development and implementation of the clinical calculator

**Fig. 10:**
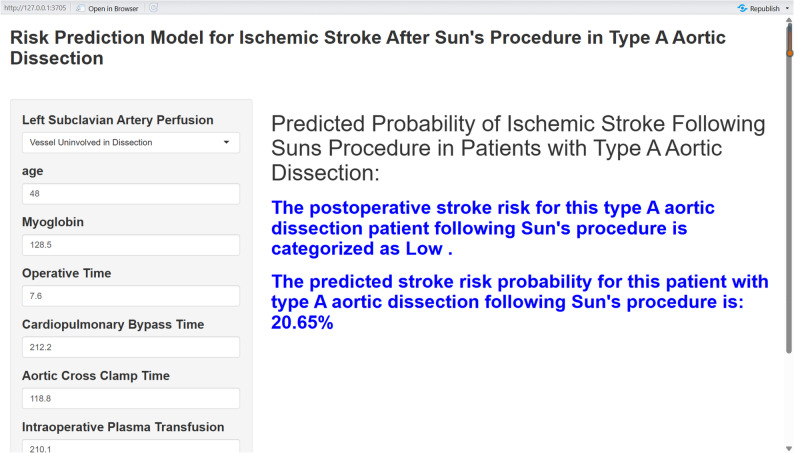
Clinical prediction model calculator

To translate the high-performance Random Forest prediction model developed in this study into a practical tool for clinical decision-making, we designed and implemented an online clinical calculator. The primary objective of this calculator is to transform the complex “black-box” machine learning model into an interactive tool with a user-friendly interface and straightforward operation. Thereby, it enables clinicians to rapidly and accurately assess the risk of postoperative ischemic stroke in individual patients at the point of care (such as during preoperative assessment or postoperative monitoring), without requiring any background in programming or statistics (Fig. 10).

## Discussion

In this study, we compared multiple machine learning models to evaluate their performance in predicting postoperative ischemic stroke in patients undergoing Sun’s procedure. Based on comprehensive analysis of multiple metrics including ROC, DCA, accuracy, sensitivity, specificity, and F1 score, the RF model was selected due to its superior overall performance and ability to effectively balance predictive accuracy with clinical utility (Table 2). These metrics highlight RF model ‘s capability to balance sensitivity and specificity, minimizing missed TAAD cases while maintaining prediction accuracy. Through Boruta algorithm and AUC value analysis, key features including cardiopulmonary bypass time, age, intraoperative blood loss, myoglobin, operative time, aortic cross-clamp time, intraoperative plasma transfusion volume and left subclavian artery perfusion were identified as significant predictors of postoperative ischemic stroke. RCS analysis revealed linear relationships and OR > 1 thresholds between these variables and postoperative ischemic stroke risk, providing valuable insights into how changes in these parameters influence clinical outcomes.

To our knowledge, this study represented the first instance of utilizing machine learning algorithms with real-world data to predict postoperative ischemic stroke in patients undergoing Sun’s procedure. However, previous studies have extensively analyzed risk factors for postoperative ischemic stroke in aortic dissection patients, many of which overlap with variables incorporated into our model. Prolonged cardiopulmonary bypass time as an independent risk factor for ischemic stroke in aortic dissection surgery has been reported in multiple studies [15]. In our findings, cardiopulmonary bypass time (OR = 1.01, 95% CI:1.00–1.02.00.02, *p* = 0.007) emerged as a risk factor for ischemic stroke, consistent with Mircea [15] and GERAADA [16] studies. Additionally, in our study RCS curve analysis revealed that when bypass time exceeded 255.1 min, ischemic stroke probability significantly increased. A study of 501 aortic arch surgery patients demonstrated that permanent neurological dysfunction correlated with prolonged operative time, while hypothermic circulatory arrest duration associated with transient neurological deficits [17]. Furthermore, the impact of cannulation strategies on postoperative ischemic stroke remains debated. Multiple studies suggest axillary artery cannulation [18–20] exhibits a trend toward reduced ischemic stroke risk by enabling antegrade cerebral perfusion during arch repair. Conversely, femoral artery cannulation introduces retrograde flow through the descending aorta, potentially mobilizing arterial embolism and increasing ischemic stroke risk [20, 21]. Our study yielded similar findings.

Age is another critical risk factor for ischemic stroke prediction. Lin et al. [22] reported a study of 1,445 TAAD patients undergoing Total Arch Replacement with Frozen Elephant Trunk (TAR + FET) surgery, finding that advanced age correlates with adverse postoperative outcomes in aortic dissection, with a mean patient age of 47.11 ± 9.99 years. Our cohort exhibited a median age of 48.00 (38.00, 55.00) years, aligning with these findings. Tamura et al. [23] conducted a retrospective TAAD study demonstrating that TAR + FET surgery is more frequently selected for individuals under 50 years. Philip et al., analyzing the international Registry of Acute Aortic Dissection(IRAD) database, revealed that TAAD patients aged ≥ 70 years face significantly increased in-hospital mortality risk from cerebrovascular events, though long-term mortality in survivors remains unaffected. Chinese ATAAD patients are notably younger than Western populations. The GERAADA study [16] reported a mean age of 61.3 ± 13.5 years, while the IRAD cohort averaged 61 ± 14.6 years [24]. Given China’s higher life expectancy and emphasis on long-term outcomes, the TAR + FET surgery has become the standardized surgical approach for ATAAD involving the entire aortic arch and descending aorta, widely adopted domestically.

Furthermore, our model incorporated several predictive indicators that have been less frequently reported in previous studies. We identified intraoperative blood loss, intraoperative plasma transfusion, and myoglobin levels as risk factors for postoperative ischemic stroke. This study found that excessive intraoperative blood loss (exceeding 2442.85 mL) contributes to the occurrence of ischemic stroke. This may be attributed to the intraoperative transfusion of packed red blood cells to maintain blood volume and the administration of certain coagulation factors for hemostasis. Numerous studies have reported that transfusion of 1–2 units of packed red blood cells is associated with an increased risk of stroke [25–27]. Martin et al. [28] reported that the use of recombinant factor VIIa (rFVIIa) in cardiac surgery patients may lead to a significant increase in stroke (OR = 3.69, 95% CI:1.1–12.38.1.38, *p* = 0.03). Similarly, Yuji Kanaoka et al. [29], in a cohort of 439 patients undergoing thoracic endovascular aortic repair (TEVAR) for aortic aneurysm, reported that an intraoperative blood loss ≥ 800 mL was an independent risk factor for cerebral infarction (OR = 24.31, *P* = 0.017).Secondly, given its superior sensitivity and specificity as a diagnostic marker, serum Troponin T has become the standard laboratory indicator for detecting myocardial injury [30]. Previous studies have demonstrated that serum Troponin T serves as a prognostic variable in both cardiac and non-cardiac surgeries, with elevated levels being associated with postoperative complications and mortality [31, 32]. Research by Piotr et al. also highlighted the utility of myocardial injury biomarkers in predicting cardiogenic shock in patients undergoing cardiac valve surgery [33]. Our findings align with and extend this evidence. We confirmed that myoglobin—a biomarker highly correlated with Troponin T—also demonstrates significant utility in diagnosing cerebral infarction following aortic dissection surgery.

In this study, the RF model demonstrated superior performance over traditional methods such as logistic regression in predicting postoperative ischemic stroke risk in patients undergoing TAR + FET surgery. While logistic regression is valued for its simplicity and interpretability, its reliance on linear assumptions and limited capacity to capture complex predictor interactions constrained its predictive performance. In contrast, the RF model achieved higher accuracy, recall, and F1 scores owing to its ability to model nonlinear relationships and complex interactions. Furthermore, SHAP analysis was employed to address the interpretability limitations of the RF model, providing clinically meaningful insights into individual predictor importance. Additionally, an interactive clinical prediction calculator was developed to enhance practical utility in clinical settings.

This study has several limitations. First, retrospective studies are prone to selection bias. Second, the lack of external validation for the model and limited dataset size restrict generalizability—external validation using multicenter data from diverse geographic regions is essential to ensure robustness and broader applicability. Collaborations with other institutions are underway to address these limitations and improve clinical utility across patient populations. Future studies should aim to resolve these limitations to further refine the model.

## Supplementary Information

Supplementary Material 1.

## Data Availability

All the data and materials are genuine and reliable.For the original data, please contact the author Siji Chen at 18319544878@163.com.
